# Accuracy of self-reported body weight, height and waist circumference in a Dutch overweight working population

**DOI:** 10.1186/1471-2288-8-69

**Published:** 2008-10-28

**Authors:** Johanna C Dekkers, Marieke F van Wier, Ingrid JM Hendriksen, Jos WR Twisk, Willem van Mechelen

**Affiliations:** 1 EMGO Institute and Department of Public and Occupational Health, VU University medical center, Van der Boechorststraat 7, 1081 BT Amsterdam, The Netherlands; 2 Body@Work, Research Center Physical Activity, Work and Health, TNO-VUmc, Amsterdam, The Netherlands; 3 Municipal Health Service The Hague, The Hague, The Netherlands; 4 TNO Quality of Life, Leiden, The Netherlands; 5 Department of Clinical Epidemiology and Biostatistics, VU University Medical Center, Amsterdam and Department of Methodology and Applied Biostatistics, Institute of Health Sciences, VU University, Amsterdam, The Netherlands

## Abstract

**Background:**

In population studies, body mass index (BMI) is generally calculated from self-reported body weight and height. The self-report of these anthropometrics is known to be biased, resulting in a misclassification of BMI status. The aim of our study is to evaluate the accuracy of self-reported weight, height and waist circumference among a Dutch overweight (Body Mass Index [BMI] ≥ 25 kg/m^2^) working population, and to determine to what extent the accuracy was moderated by sex, age, BMI, socio-economic status (SES) and health-related factors.

**Methods:**

Both measured and self-reported body weight and body height were collected in 1298 healthy overweight employees (66.6% male; mean age 43.9 ± 8.6 years; mean BMI 29.5 ± 3.4 kg/m^2^), taking part in the ALIFE@Work project. Measured and self-reported waist circumferences (WC) were available for a sub-group of 250 overweight subjects (70.4% male; mean age 44.1 ± 9.2 years; mean BMI 29.6 ± 3.0 kg/m^2^). Intra Class Correlation (ICC), Cohen's kappa and Bland Altman plots were used for reliability analyses, while linear regression analyses were performed to assess the factors that were (independently) associated with the reliability.

**Results:**

Body weight was significantly (p < 0.001) under-reported on average by 1.4 kg and height significantly (p < 0.001) over-reported by 0.7 cm. Consequently, BMI was significantly (p < 0.001) under-reported by 0.7 kg/m^2^. WC was significantly (p < 0.001) over-reported by 1.1 cm. Although the self-reporting of anthropometrics was biased, ICC's showed high concordance between measured and self-reported values. Also, substantial agreement existed between the prevalences of BMI status and increased WC based on measured and self-reported data. The under-reporting of BMI and body weight was significantly (p < 0.05) affected by measured weight, height, SES and smoking status, and the over-reporting of WC by age, sex and measured WC.

**Conclusion:**

Results suggest that self-reported BMI and WC are satisfactorily accurate for the assessment of the prevalence of overweight/obesity and increased WC in a middle-aged overweight working population. As the accuracy of self-reported anthropometrics is affected by measured weight, height, WC, smoking status and/or SES, results for these subgroups should be interpreted with caution. Due to the large power of our study, the clinical significance of our statistical significant findings may be limited.

**Trial Registration:**

ISRCTN04265725

## Background

The high and still increasing prevalence of overweight (Body Mass Index [BMI] ≥ 25 kg/m^2^) and obesity (BMI ≥ 30 kg/m^2^) seriously threaten public health worldwide. Overweight and obesity are associated with multiple health problems [1] and with excess mortality [1-3].

The Body Mass Index (BMI) is the most commonly used measure of overweight or general adiposity, and is calculated as body weight (in kg) divided by squared body height (in meters). Consequently, knowledge on body weight and height in a population is relevant to be able to assess the prevalence of overweight and obesity, and to identify subgroups that are at increased risk to develop overweight and obesity-related health problems and to die prematurely.

As self-measurement of body weight and height is simple and inexpensive, it is a suitable method to collect data from a large number of individuals [4]. Previous studies have shown that adult people tend to under-report their body weight and to over-report their body height [5-12], especially those with increased weight [7,10-12]. In addition, self-reported anthropometrics are more biased in older than in younger subjects [5]. Inaccurate measurements of body weight and height will lead to biased calculations of BMI, and consequently to an inaccurate assessment of the disease and mortality risk of a population.

Waist circumference (WC) is a measure of abdominal or central adiposity. As abdominal fat is a better predictor of risk for obesity-related disorders than general adiposity[13], it may be a more useful clinical tool to identify the prevalence of overweight and obesity and their related risk factors than BMI. Therefore, it is important to know whether WC can be accurately self-reported. In the limited number of studies that have been performed on the accuracy of self-reported WC in adults [14-22], underreporting of WC in both men and women has been the most consistent finding. Only two studies assessed the accuracy of self-reported WC as well as that of body weight and height in the same population [18,20], of which one included only women [18].

The prevalence of overweight is known to vary with socio-economic status (SES) [23]. Obesity has been reported to be inversely associated with SES, especially in women. The extent of misreporting anthropometrics seems to be greater in persons with a higher body weight or BMI [18,21]. Therefore, it is conceivable that the prevalence of misreporting in those from a low SES will be higher than in those from a higher SES. As yet, the limited number of studies investigating the effect of SES on the accuracy of self-reporting body weight and height has been inconclusive. Both an effect [6,7,24,25] and no effect [26] of SES on the accuracy of self-reported body weight and height has been found. Regarding self-reported WC, the few studies addressing this issue found no effect of SES on the misreporting of WC [14,17,20].

Several studies on the accuracy of self-reported anthropometrics have included health-related factors that may be associated with the accuracy, such as smoking status [6,26], physical activity level [12,26], adhering to a special diet [26], weight history [6], and medication for cardiovascular risk factors [7]. It is conceivable that the number of attempts to loose weight and frequency of weighing oneself may also be associated with the accuracy of self-reported anthropometrics, but to our knowledge these associations have not been investigated previously.

The accuracy of self-reported weight, height and WC has never been studied in a Dutch population, neither in relation to SES or health-related factors. The main objective of this study was to evaluate the accuracy of self-reported body weight, height and waist circumference among a Dutch overweight working population. A secondary objective was to assess to what extent the accuracy was affected by sex, age, overweight status, SES and the health-related factors smoking status, medication use, frequency of weighing oneself and number of attempts to loose weight.

## Methods

### Subjects

Subjects were 1298 overweight employees, taking part in the ALIFE@Work project. The ALIFE@Work project is an ongoing randomized controlled trial, in which a lifestyle intervention program aimed at changing physical activity and nutrition in an overweight working population is being evaluated [27].

For these 1298 subjects (66.6% male; mean BMI 29.5 ± 3.4 kg/m^2^; mean age 43.9 ± 8.6 years), both measured and self-reported body weight and body height measures were available at the start of the study. Self-reported WC was available for 1276 of the 1298 participants, and measured WC only for a random sub-sample of 250 participants. Consequently, both measured and self-reported WC were available for 250 subjects (70.4% male; mean BMI 29.6 ± 3.0 kg/m^2^; mean age 44.1 ± 9.2 years).

The Medical Ethical Committee of the VU University medical center reviewed and approved the study. Written informed consent was obtained from all participants. All subjects participated voluntarily and were free to cancel their participation, without reason, at any time throughout the course of the study.

### Study design and procedures

Eligible subjects (i.e., BMI ≥ 25 kg/m^2^) who agreed to participate in ALIFE@Work were invited to have their body weight and body height measured. All eligible subjects had been sent an information brochure on the project, upon which they could decide to participate in the study. Right after these measurements, a randomization allocated a sub-sample of 20% to a group receiving additional biological and anthropometric measurements, including WC. These additional measurements took place within two weeks after the assessment of body weight and height. Because of limited resources, it was not feasible to do these additional assessments for all study subjects. All measurements were done by the same two trained researchers according to standardized protocols, at or near the employee's work site.

Together with the invitation to have their body weight and height measured, participants received a questionnaire at home, approximately two weeks before the measurement took place, in which they were asked, among other questions, to report their body weight, height and waist circumference. The questionnaire also asked, among other issues, about their education level and health-related characteristics. Subjects were asked to bring the questionnaire with them to the baseline measurement appointment or sent it back in a pre-stamped envelope. A detailed description of the study design has been published elsewhere [27].

### Anthropometrics

#### Measured body weight and height

Body weight (in kg to the nearest 0.1 kg) was measured with a reliable weighing scale (Seca 770; Seca GmbH & Co, Hamburg, Germany) while participants were wearing light clothing and no shoes. Body height (in cm to the nearest 0.1 cm) was measured with a portable wall-mounting height scale with a measuring slide and a heel plate (Seca 214, Leicester Height Measure; Seca GmbH & Co, Hamburg, Germany). Position of the head was standardized by asking the subject to stand straight, without shoes and with the heels together. Height and weight were measured twice without delay between the measurements, and for both the mean value of the two measurements was taken. Next, the BMI was calculated from these averaged values. Based on BMI, subjects were classified as being overweight (25 kg/m^2 ^≤ BMI <30 kg/m^2^; N = 861 [66.3%]) or as being obese (BMI ≥ 30 kg/m^2^; N = 437 [33.7%]).

#### Self-reported body weight and height

Besides the objectively measured weight and height, participants were asked to report their body weight and height in a questionnaire that was sent to their home about two weeks before the measurement took place. Consequently, at time of the self-reporting, they were not (yet) aware of their measured body weight and height. Self-reported body weight was collected with the question: "What is your current body weight?" in kg with the accompanying text "Do weigh your self by preference in the morning before breakfast in underwear or light clothing (round to 0.5 kg)". Self-reported height was obtained with the question: "What is your height?" in cm.

#### Measured waist circumference

WC (in cm), as a measure of central adiposity, was measured twice to the nearest 0.1 cm with a measuring tape (Gulick; Creative Health Products, Ann Arbor, MI, USA) at the midpoint between the lower border of the ribs and the upper border of the pelvis. Next, the two measurements were averaged. The two measurements were taken right after each other, without delay.

#### Self-reported waist circumference

In addition, employees were asked to report their WC by answering the question: "What is your waist circumference?" in cm. The accompanying sentence read: "Use the tape measure and instructions that were sent to you along with the questionnaire". A non-stretchable paper measuring tape (range 0–135), which was especially produced for the study, and measuring instructions for use were sent to all participants along with the questionnaire. Subjects were instructed to measure their WC twice without delay between measurements (to the nearest 0.5 cm) at the midpoint between the lower border of the ribs and the upper border of the pelvis, on bare skin with clothing removed, during exhalation, while standing straight-up with the legs 25 to 30 cm apart. They were explicitly instructed to perform the measurement themselves and not having it done by someone else. To ensure proper assessment of the midpoint between the lower border of the ribs and the upper border of the pelvis, subjects were asked to first localize these body points and to mark them on skin with a pen. They were instructed to hold the measuring tape in horizontal position while measuring. Next, they were instructed to average the two readings, and to report the averaged value. Researchers followed the same instructions when measuring the WC. Participants and researchers were not aware of each others measurement outcomes.

According to National Institutes of Health cutoff points, males with a WC of 102 cm or higher and females with one of 88 cm or higher are considered to have an increased WC, placing them at increased risk to develop several health problems [11]. Therefore, for both males and females two groups were created based on their measured WC resulting in groups with normal (males, N = 76 [30.4%]; females, N = 20 [8%]) and with increased WC (males, N = 100 [40%]; females, N = 54 [21.6%]). When these groups were created based on their self-reported WC, 28% of the males (N = 70) and 6.8% of the females (N = 17) had a normal waist and 42.2% males (N = 106) and 22.8% of the females (N = 57) an increased WC.

### Socio-economic status

SES was represented by education level. Subjects were asked to indicate their highest education level on an ordering 6-point scale, ranging from no education to postgraduate education (1 'no education' [0.2%]; 2 'primary' [0.4%]; 3 'lower vocational' [4.3%]; 4 'medium vocational' [34.6%]; 5 'upper vocational' [47.2%]; 6 'university/postgraduate' [13.3%]). Next, education level was divided into two categories: low education level (N = 513; no education to medium vocational level) and high education level (N = 784; upper vocational level to postgraduate education level). The low and high education level groups were perceived as low and high SES groups, respectively. Education level was missing for one subject.

### Health-related characteristics

Apart from measured body weight, height and BMI, age and SES, health-related variables considered to affect the bias were also studied. These variables included: medication use for overweight-related health complaints, smoking status, number of attempts to loose weight and the frequency of weighing oneself.

Information on the use of medication for overweight-related health conditions was obtained by the question: 'Do you use medication for one of the following health conditions: hypercholesterolaemia, hypertension, diabetes, depression, myocardial infarct, angina pectoris, stroke?' (0 'no', N = 1031 [79.4%]; 1 'yes', N = 218 [16.8%]). Information on medication use was missing for 49 subjects.

Smoking status was assessed by the question: 'Do you smoke cigarettes, shag, cigars or pipe?' (0 'no', N = 1103 [85.1%]; 1 'yes', N = 193 [14.9%]). There were two missings for smoking status.

Frequency of weighing oneself was obtained by the question: 'How often do you weigh yourself?' (0 'never', N = 61 [4.7%]; 1 'once a year or less', N = 81 [6.2%]; 2 'every other month', N = 219 [16.9%]; 3 'monthly', N = 178 [13.7%]; 4 'every two weeks', N = 132 [10.2%]; 5 'weekly', N = 428 [33%]; 6 'daily', N = 194 [14.9%]; 7 'more than once a day', N = 5 [0.4%]). Frequency of weighing oneself was divided into a group with a low frequency of weighing oneself (0 'every two weeks or less', N = 671 [51.7%]) and a group with a high frequency of weighing oneself (1 'weekly or more often', N = 627 [48.3%]).

Information on the number of attempts to loose weight was obtained by the following question: 'How often did you try loosing weight in the past two years?' (0 'never', N = 417 [32.1%]; 1 'once', N = 304 [23.4%]; 2 'two to three times', N = 301 [23.2%]; 3 'four to five times', N = 37 [2.9%]; 4 'more than five times', N = 40 [3.1%]; 5 'continuously', N = 196 [15.1%]). The variable was divided into three categories: 'no attempt' (N = 420 [32.4%]) 'one to three attempts' (N = 605 [46.6%]) and 'more than 4 attempts' (N = 273 [21%]). Number of attempts to loose weight was missing for three subjects.

### Statistics

Reliability between measured and self-reported values of continuous variables was evaluated with the use of Intra Class Correlation Coefficient (ICC) and 95% Confidence Interval (95% CI). To assess the agreement between measured and self-reported prevalence of overweight and increased WC Cohen's kappa was used. The strength of the agreement was classified as suggested by Landis and Koch [28]. The percentage of agreement was calculated as well.

In addition, Bland and Altman [29] plots were used in order to examine the individual agreement between self-reported and measured anthropometrics. In these plots, the differences between measured and self-measured values (measured minus self-reported) were plotted against the mean of measured and self-reported values. Limits of agreement were calculated as the mean difference ± 1.96 standard deviations (SD). Paired t-tests were used to assess statistically significant differences between measured and self-reported values.

Regression analysis was used to evaluate what variables were (independently) associated with the difference between measured and self-reported anthropometrics. Separate models were built for bias in the self-report of body weight, height, BMI and WC. The following variables were included in the regression models as independent variables: sex, age, SES, measured weight, measured height, measured BMI, measured WC, smoking status, medication use, frequency of weighing oneself and number of attempts to loose weight. For the latter variable, two dummy variables were created and coded such that the 'one to three attempts' and 'more than four attempts' groups were compared with the 'no attempt' group.

All reliability analyses were performed for all subjects, as well as for sex (864 males, 434 females), BMI groups (overweight [N = 861], obese [N = 437]), SES groups (low [N = 514], high [N = 782]), age groups (low [N = 649], high [N = 649]), smoking status (smoking [N = 193], non-smoking [N = 1103]), medication use (use [N = 218], no use [N = 1031]) and frequency of weighing oneself (low frequency [N = 671], high frequency [N = 627]). A median split for age had yielded a younger age group (age ≤ 44.5 yrs, mean age 36.7 ± 5.2 yrs, N = 649) and an older age group (age > 44.5 yrs, mean age 51.1 ± 4.3 yrs, N = 649).

All analyses were performed using SPSS software (version 12.0.1) and p-values <0.05 were considered to be significant.

## Results

### Reliability of self-reported body weight, height, BMI and WC

The average intra-class correlation coefficients for body weight, height, BMI and WC demonstrated high concordance between measured and self-reported measures (Table 1). Comparably high average intra-class correlation coefficients were found for the anthropometric measures in the different subgroups (ICC range males: 0.96–0.99; females: 0.91–0.99; low age group: 0.96–0.99; high age group: 0.95–1.00; overweight: 0.92–0.99; obese: 0.95–0.99; low SES: 0.95–1.00; high SES: 0.96–1.00; smoking: 0.93–0.99; non-smoking: 0.96–1.00; medication use: 0.98–1.00; no medication use:0.95–1.00; low frequency of weighing oneself: 0.96–0.99; high frequency of weighing oneself: 0.95–1.00) (see Additional file 1: Average intra-class correlation coefficients (95% CI) by sex, and by age, BMI groups, SES groups, smoking status, medication use and frequency of weighing oneself groups).

**Table 1:** Average intra-class correlation coefficients (95% CI) for body weight, height, BMI and waist circumference in all 1298 subjects

Anthropometrics	ICC	95% CI
Body weight (kg)	1.00	0.99 to 1.00
Body height (cm)	0.99	0.99 to 0.99
BMI (kg/m^2^)	0.99	0.98 to 0.99
WC (cm)*	0.96	0.94 to 0.97

BMI = Body Mass Index; WC = waist circumference

* Results on WC are based on a sub-sample of 250 subjects (176 males and 74 females)

Body weight (p < 0.001) and height (p < 0.001) were respectively, significantly under-reported by 1.4 kg and over-reported by 0.7 cm (Table 2). This resulted in BMI being under-reported by 0.7 kg/m^2 ^(p < 0.001). WC was significantly (p < 0.001) over-reported by 1.1 cm (Table 2).

**Table 2:** Mean (SD) measured and self-reported anthropometrics, the mean differences (SD) and the p-value of the paired t-test for all 1298 subjects

Anthropometrics	Measured	Self-reported	Difference	p-value
Body weight (kg)	92.7 (14.0)	91.3 (13.8)	1.4 (1.9)	<0.001
Body height (cm)	177.0 (9.1)	177.7 (9.2)	-0.7 (1.5)	<0.001
BMI (kg/m^2^)	29.5 (3.4)	28.9 (3.3)	0.7 (0.8)	<0.001
WC (cm)*	101.8 (9.7)	102.9 (9.8)	-1.1 (4.0)	<0.001

* Results on WC are based on a sub-sample of 250 subjects (176 males and 74 females)

As a consequence of the misreporting, the prevalences of overweight, obesity and WC were, respectively, over-reported by 3.4%, under-reported by 6.9% and over-reported by 3.6% (Table 3). The overall prevalence of overweight (overweight and obesity combined) was under-reported by 3.5% (not shown). The percentages agreement between self-reported and measured prevalence of overweight and obesity and of increased WC were substantial (overweight/obese: 91.4%, kappa 0.80; WC: 86.8%, kappa 0.72).

**Table 3:** Prevalences (%) of overweight, obesity and increased WC based on measured and self-reported values for all 1298 subjects

	All subjects (N = 1298)
Overweight measured	66.3
Overweight self-reported	69.7
	
Obesity measured	33.7
Obesity self-reported	26.8
	
Increased WC measured*	61.6
Increased WC self-reported*	65.2

* Results on WC are based on a sub-sample of 250 subjects (176 males and 74 females)

**Figure 1:**
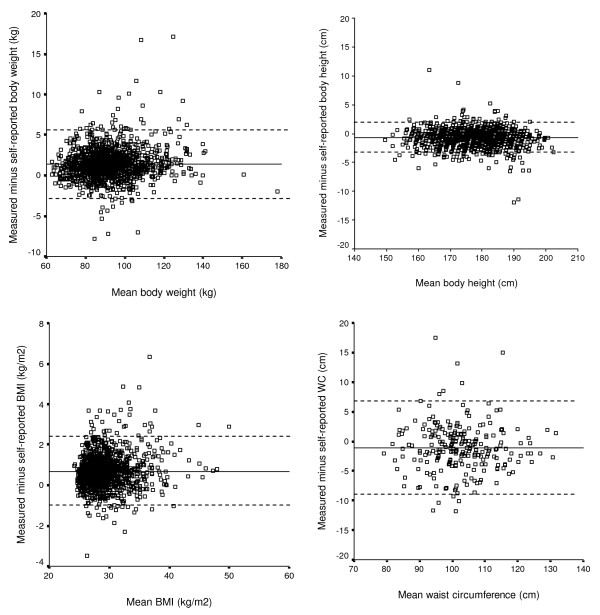
**a-d. Bland-Altman plots of the difference between measured and self-reported body weight (a), height (b), BMI (c) and WC (d) plotted against the mean**. In each figure, the solid line represents the mean difference between the measured and self-reported value (body weight: 1.4 kg; height: -0.7 cm; BMI: 0.7 kg/m^2^; WC: -1.1 cm) and the dashed lines represent the 95% limits of agreement (body weight -2.4, 5.2; body height -3.7, 2.3; BMI -0.9, 2.3; WC -8.9, 6.8).

Figure 1a–d shows the extent of misreporting of body weight, height, BMI and WC. It can be observed that there were individual differences in the accuracy of self-reported anthropometrics. For example, the difference between measured and self-reported values of WC ranged from -8.9 cm (over-reporting) to 6.8 cm (under-reporting).

### Accuracy of self-reported anthropometrics in relation to sex, BMI status, age, SES and health-related characteristics

Additional analyses were performed to assess the accuracy of self-reported anthropometrics in relation to sex (male/female), BMI status (overweight/obese), age (low/high), SES (low/high), medication use for overweight-related health conditions (use/no use), smoking status (smoking/non-smoking) and frequency of weighing oneself (low/high). In all subgroups, body weight was significantly under-reported and body height significantly over-reported, resulting in BMI being significantly (p's<0.001) under-reported (Table 4).

**Table 4:** Mean (SD) measured and self-reported anthropometrics, the mean differences (SD) and p-values by sex, age, BMI status, SES, smoking status, medication use and frequency of weighing oneself

Anthropometrics	Measured	Self-reported	Difference	p-value	Measured	Self-reported	Difference	p-value
	**Males (N = 864)**				**Females (N = 434)**			
Body weight (kg)	96.8 (12.7)	95.4 (12.6)	1.5 (2.0)	<0.001	84.5 (12.7)	83.3 (12.4)	1.2 (1.8)	<0.001
Body height (cm)	181.5 (6.7)	182.2 (6.7)	-0.7 (1.6)	<0.001	168.2 (6.3)	168.9 (6.3)	-0.7 (1.5)	<0.001
BMI (kg/m^2^)	29.4 (3.1)	28.7 (3.0)	0.7 (0.8)	<0.001	29.9 (4.0)	29.2 (3.9)	0.7 (0.8)	<0.001
Waist (cm)*	104.5 (8.5)	105.9 (8.5)	-1.4 (3.3)	<0.001	95.3 (9.5)	95.5 (8.9)	-0.3 (5.2)	0.66

	**Low age (N = 649)**				**High age (N = 649)**			
Body weight (kg)	92.9 (14.5)	91.5 (14.2)	1.3 (2.0)	<0.001	92.6 (13.4)	91.1 (13.3)	1.5 (1.8)	<0.001
Body height (cm)	176.7 (9.5)	177.4 (9.6)	-0.7 (1.4)	<0.001	177.3 (8.6)	178.0 (8.8)	-0.7 (1.6)	<0.001
BMI (kg/m^2^)	29.7 (3.6)	29.0 (3.5)	0.7 (0.9)	<0.001	29.4 (3.2)	28.7 (3.1)	0.7 (0.8)	<0.001
Waist (cm)*	100.7 (10.3)	102.4 (10.2)	-1.7 (3.9)	<0.001	102.9 (9.1)	103.3 (9.4)	-0.4 (4.0)	0.21

	**Overweight (N = 861)**				**Obese (N = 437)**			
Body weight (kg)	87.1 (9.6)	85.9 (9.6)	1.3 (1.7)	<0.001	103.8 (14.6)	102.1 (14.4)	1.7 (2.3)	<0.001
Body height (cm)	177.3 (8.8)	177.9 (9.0)	-0.6 (1.5)	<0.001	176.5 (9.6)	177.4 (9.7)	-0.9 (1.6)	<0.001
BMI (kg/m^2^)	27.6 (1.3)	27.1 (1.4)	0.6 (0.7)	<0.001	33.2 (3.3)	32.4 (3.3)	0.9 (1.0)	<0.001
Waist (cm)*	97.8 (7.2)	98.9 (7.2)	-1.1 (3.9)	<0.001	109.3 (9.5)	110.2 (9.9)	-0.9 (4.2)	<0.05

	**Low SES (N = 513)**				**High SES (N = 784)**			
Body weight (kg)	94.0 (14.7)	92.3 (14.4)	1.7 (2.0)	<0.001	91.9 (13.4)	90.7 (13.3)	1.2 (1.8)	<0.001
Body height (cm)	176.6 (9.2)	177.4 (9.4)	-0.8 (1.6)	<0.001	177.3 (9.0)	178.0 (9.1)	-0.7 (1.5)	<0.001
BMI (kg/m^2^)	30.1 (3.7)	29.3 (3.6)	0.8 (0.9)	<0.001	29.2 (3.2)	28.6 (3.2)	0.6 (0.8)	<0.001
Waist (cm)*	102.1 (9.6)	102.6 (9.8)	-0.5 (4.3)	0.28	101.6 (9.9)	103.0 (9.9)	-1.5 (3.8)	<0.001

	**Smoking (N = 193)**				**Non-smoking (N = 1103)**			
Body weight (kg)	93.8 (14.4)	92.5 (14.2)	1.2 (2.2)	<0.001	92.6 (13.9)	91.1 (1.9)	1.4 (1.9)	<0.001
Body height (cm)	176.4 (9.3)	177.2 (9.4)	-0.8 (1.4)	<0.001	177.1 (9.0)	177.8 (9.2)	-0.7 (1.6)	<0.001
BMI (kg/m^2^)	30.1 (3.5)	29.4 (3.5)	0.7 (0.9)	<0.001	29.4 (3.4)	28.8 (3.3)	0.7 (0.8)	<0.001
Waist (cm)*	101.1 (9.9)	102.4 (9.9)	-1.3 (5.0)	0.13	101.9 (9.8)	102.9 (9.9)	-1.0 (3.8)	<0.001

	**Medication use (N = 218)**				**No medication use (N = 1031)**			
Body weight (kg)	95.6 (15.6)	94.0 (15.4)	1.6 (2.1)	<0.001	91.9 (13.4)	90.6 (13.2)	1.4 (1.9)	<0.001
Body height (cm)	176.8 (8.6)	177.6 (8.7)	-0.9 (1.4)	<0.001	177.0 (9.2)	177.7 (9.3)	-0.7 (1.6)	<0.001
BMI (kg/m^2^)	30.5 (3.8)	29.7 (3.7)	0.8 (0.9)	<0.001	29.3 (3.3)	28.6 (3.2)	0.6 (0.8)	<0.001
Waist (cm)*	105.2 (11.3)	106.6 (10.5)	-1.4 (3.4)	0.02	101.3 (9.5)	102.3 (9.7)	-1.0 (4.1)	<0.001

	**Low frequency of weighing oneself (N = 671)**				**High frequency of weighing oneself (N = 627)**			
Body weight (kg)	94.4 (14.1)	92.9 (13.9)	1.4 (2.2)	<0.001	91.0 (13.6)	89.6 (13.5)	1.4 (1.6)	<0.001
Body height (cm)	178.1 (9.0)	178.8 (9.2)	-0.7 (1.6)	<0.001	175.9 (9.0)	176.6 (9.1)	-0.7 (1.5)	<0.001
BMI (kg/m^2^)	29.7 (3.4)	29.0 (3.2)	0.7 (0.9)	<0.001	29.4 (3.5)	28.7 (3.4)	0.7 (0.7)	<0.001
Waist (cm)*	104.1 (9.4)	105.1 (9.2)	-1.1 (3.6)	<0.001	99.4 (9.6)	100.5 (10.0)	-1.1 (4.4)	<0.01

* Results on WC are based on a sub-sample of 250 subjects (176 males and 74 females; low age group [N = 125], high age group [N = 125]; low BMI group [N = 163], high BMI group [N = 87]; low SES group [N = 99], high SES group [N = 151]; smoking [N = 36], non-smoking [N = 212]; medication use [N = 33], no medication use [N = 209]; low frequency of weighing [N = 125], high frequency of weighing [N = 123]).

As a consequence of the misreporting of BMI, the prevalence of overweight was over-reported and that of obesity under-reported (Table 5). The overall prevalence of overweight (overweight and obesity combined) was under-reported in all subgroups, except for the BMI groups (not shown). For example, in males the prevalence of overweight was over-reported by 4.1%, the prevalence of obesity under-reported by 6.8% and the overall prevalence of overweight under-reported by 2.7% (6.8 minus 4.1). The over-reporting of the prevalence of overweight in the subgroups is due to the fact that obese subjects who under-report their BMI status automatically fall into the overweight category.

**Table 5:** Prevalences (%) of overweight, obesity and increased WC based on measured and self-reported values for sex, age, BMI status, SES, smoking status, medication use and frequency of weighing oneself

	**Males (N = 864)**	**Females (N = 434)**
Overweight measured	68.1	62.9
Overweight self-reported	72.2	64.7
Obesity measured	31.9	37.1
Obesity self-reported	25.1	30.2
Increased WC measured^1^	56.8	73.0
Increased WC self-reported^1^	60.2	77.0
	**Low age (N = 649)**	**High age (N = 649)**
Overweight measured	64.4	68.3
Overweight self-reported	66.3	73.2
Obesity measured	35.6	31.7
Obesity self-reported	29.1	24.5
Increased WC measured^1^	59.2	64.0
Increased WC self-reported^1^	66.4	64.0

	**Overweight (N = 861)**	**Obese (N = 437)**
Overweight measured	100.0	0.0
Overweight self-reported^2^	93.5	22.9
Obesity measured	0.0	100.0
Obesity self-reported	1.3	77.1
Increased WC measured^1^	44.2	94.3
Increased WC self-reported^1^	49.7	94.3

	**Low SES (N = 513)**	**High SES (N = 784)**
Overweight measured	61.0	69.8
Overweight self-reported	66.7	71.7
Obesity measured	39.0	30.2
Obesity self-reported	31.2	24.0
Increased WC measured^1^	68.7	57.0
Increased WC self-reported^1^	68.7	62.9

	**Smoking (N = 193)**	**Non-smoking (N = 1103)**
Overweight measured	59.6	67.5
Overweight self-reported	63.7	70.9
Obesity measured	40.4	32.5
Obesity self-reported	34.7	25.4
Increased WC measured^1^	41.7	39.2
Increased WC self-reported^1^	47.2	41.0

	**Medication use (N = 218)**	**No medication use (N = 1031)**
Overweight measured	54.6	69.0
Overweight self-reported	61.5	71.5
Obesity measured	45.4	31.0
Obesity self-reported	36.7	24.5
Increased WC measured^1^	60.6	36.8
Increased WC self-reported^1^	54.5	40.7

	**Low frequency of weighing oneself (N = 671)**	**High frequency of weighing oneself (N = 627)**
Overweight measured	63.9	68.9
Overweight self-reported	69.4	70.0
Obesity measured	36.1	31.1
Obesity self-reported	28.0	25.5
Increased WC measured^1^	51.2	27.6
Increased WC self-reported^1^	52.0	31.7

^1 ^Results on WC are based on a sub-sample of 250 subjects (176 males and 74 females; low age group [N = 125], high age group [N = 125]; low BMI group [N = 163], high BMI group [N = 87]; low SES group [N = 99], high SES group [N = 151]; smoking [N = 36], non-smoking [N = 212]; medication use [N = 33], no medication use [N = 209]; low frequency of weighing [125], high frequency of weighing [123]).

^2 ^45 subjects (5.2%) had a healthy weight based on their self-reported BMI

Regarding the BMI groups (i.e., overweight and obese groups), in the overweight group the prevalence of overweight was under-reported by 6.5% and in the obese group the prevalence of obesity was under-reported by 22.9% (Table 5).

The percentage of agreement between self-reported and measured BMI status was substantial to almost perfect in all groups (males: 91.6%, kappa 0.79; females: 91.2%, kappa 0.81; low age group: 92.3%, kappa 0.83; high age group: 90.6%, kappa 0.77; low SES group: 90.3%, kappa 0.79; high SES group: 92.2%, kappa 0.80; smoking: 91.2%, kappa 0.81; non-smoking: 91.5%, kappa 0.79; medication use: 89.4%, kappa 0.78; no medication use: 91.8%, kappa 0.80; low frequency of weighing: 78%, kappa 0.78; high frequency of weighing: 92.8%, kappa 0.82).

WC was significantly over-reported in all subgroups, except in females, high age subjects, low SES subjects and smoking subjects (Table 4). This led to the prevalence of increased WC to be over-reported in all subgroups, except in the high age, obese, low SES and medication use subgroups (Table 5).

The percentage of agreement between self-reported and measured increased WC was substantial in all groups (males: 86.4%, kappa 0.72; females: 87.7%, kappa 0.68; low age: 84.8%, kappa 0.68; high age: 88.8%, kappa 0.72; overweight: 81.0%, kappa 0.62; obese: 97.7%, kappa 0.79; low SES: 89.9%, kappa 0.77; high SES: 84.8%, kappa 0.68; medication use: 93.9%, kappa 0.80; no medication use: 92.8%, kappa 0.78; smoking: 94.4%, kappa 0.89; non-smoking: 98.6%, kappa 0.78; low frequency of weighing: 88.0%, kappa 0.76; high frequency of weighing: 92.7%, kappa 0.83).

Univariate regression analyses showed that sex (p < 0.05), measured weight (p < 0.001), BMI status (p < 0.001), SES (p < 0.001) and medication use (p < 0.01) significantly affected the difference between measured and self-reported body weight, height and/or BMI (Table 6). Males, obese and low SES subjects under-reported their body weight significantly more than females, overweight and high SES subjects, respectively (on average 0.25 kg, 0.49 kg and 0.46 kg more, respectively). Obese subjects also significantly (p < 0.001) over-reported their body height on average 0.3 cm more than overweight subjects. The underreporting of BMI status was significantly (p < 0.001) greater in obese subjects, low SES subjects (p < 0.001) and subjects using medication (p < 0.01) compared to, respectively, overweight, high SES subjects and subjects using no medication. Also, heavier subjects under-reported their body-weight and BMI status and over-reported their height to a significantly (p < 0.001) greater extent than less heavy subjects.

**Table 6:** Results (regression coefficients [b] and 95% confidence intervals [95% CI]) of univariate regression analyses for sex, age, BMI status, SES, measured weight, measured height, measured WC and health-related factors in relation to differences (bias) between measured and self-reported BMI, body weight, body height and WC

	Bias BMI		Bias body weight		Bias body height		Bias WC	
	b	95% CI	b	95% CI	b	95% CI	b	95% CI

Sex	-0.01	-0.11, 0.08	0.25*	0.03, 0.47	0.02	-0.16, 0.20	-1.13*	-2.21, -0.04
Age	0.03	-0.06, 0.12	0.14	-0.07, 0.35	0.03	-0.14, 0.20	1.23*	0.24, 2.21
BMI status	0.30*‡*	0.21, 0.39	0.49*‡*	0.26, 0.71	-0.30*‡*	-0.47, -0.12	0.25	-0.79, 1.30
SES	-0.20*‡*	-0.28, -0.10	-0.46*‡*	-0.67, -0.25	0.09	-0.08, 0.27	-0.93	-1.94, 0.09
Measured weight (kg)	0.01*‡*	0.01, 0.01	0.02*‡*	0.02, 0.03	-0.01*‡*	-0.01, -0.00	0.01	-0.04, 0.03
Measured height (m)	-0.00	-0.01, 0.00	0.01	-0.00, 0.02	0.00	-0.01, 0.01	-4.35	-9.78, 1.07
Measured WC (cm)							0.08*†*	0.02, 0.13
Smoking status	-0.04	-0.16, 0.09	-0.21	-0.50, 0.09	-0.07	-0.30, 0.17	-0.27	-1.69, 1.15
Medication use	0.16*†*	0.04, 0.28	0.26	-0.02, 0.54	-0.20	-0.43, 0.02	-0.45	-1.94, 1.03
Frequency of weighing	-0.03	-0.12, 0.06	-0.05	-0.26, 0.16	0.08	-0.09, 0.25	0.03	-0.97, 1.02
Losing weight attempts								
1 to 3 attempts	0.05	-0.06, 0.15	0.13	-0.12, 0.37	0.05	-0.14, 0.24	-0.30	-1.49, 0.89
≥ 4 attempts	0.09	-0.04, 0.21	-0.10	-0.40, 0.19	-0.18	-0.41, 0.06	0.93	-0.45, 2.31

*†‡ significant different from the estimate of the reference group (i.e., females, low age, overweight, low SES, non-smoking, no medication use, low frequency of weighing oneself, no attempts to loose weight) or from 0 for measured body weight, height, and WC (* p < 0.05; † p < 0.01; ‡ p < 0.001)

The multivariate regression analyses showed that measured weight, height and SES were significantly (p < 0.01) independently associated with differences between measured and self-reported body weight and BMI status (Table 7). Smoking turned out to be a significant predictor (p < 0.05) of the difference between measured weight and self-reported weight as well, with non-smoking subjects under-reporting their body weight to a greater extent than smoking subjects. BMI status was a significant (p < 0.01) independent predictor of the bias in the self-reporting of height with obese subjects over-reporting their height to a greater extent than overweight subjects.

**Table 7:** Results (regression coefficients [b] and 95% confidence intervals [95% CI]) of multivariate regression analyses for sex, age, BMI status, SES, measured weight, measured height, measured WC and health-related factors in relation to differences (bias) between measured and self-reported BMI, body weight, body height and WC^1^

	Bias BMI		Bias body weight		Bias body height		Bias WC	
	b	95% CI	b	95% CI	b	95% CI	b	95% CI

Intercept	2.80	1.89, 3.71	2.73	0.57, 4.89	-0.61	-0.71, -0.50	-39.13	-56.13, -22.12
Sex							-3.35*‡*	-4.84, -1.86
BMI status					-0.30*†*	-0.47, -0.12		
SES	-0.14*†*	-0.23, -0.05	-0.39*‡*	-0.60, -0.18				
Measured weight (kg)	0.02*‡*	0.01, 0.02	0.03*‡*	0.02, 0.04			-0.25*‡*	-0.34, -0.16
Measured height (m)	-0.02*‡*	-0.03, -0.01	-0.02*†*	-0.04, -0.01			15.08*†*	5.15, 25.01
Measured WC (cm)							0.36*‡*	0.26, 0.46
Smoking			-0.30*	-0.60, -0.01				

^1 ^Only the significant variables are being displayed;* p < 0.05; † p < 0.01; ‡ p < 0.001

Measured WC, sex and age were significantly (p < 0.05) associated with the difference between measured and self-reported WC, indicating that males, younger subjects and subjects with a lower measured WC over-reported their WC to a significantly greater extent than females, older subjects and subjects with a larger measured WC (Table 6). Measured WC and sex remained significantly (p < 0.001) associated with the difference between measured and self-reported WC in the multivariate analysis (Table 7). In addition, measured weight (p < 0.001) and measured height (p < 0.01) turned out to be independently associated with the difference between measured and self-reported WC, suggesting heavier subjects and less tall subjects to over-report their WC more than less heavy and taller subjects.

## Discussion

This study aimed to evaluate the accuracy of self-reported body weight, height and WC in an overweight working population and to assess whether accuracy was affected by sex, age, BMI, SES and health-related characteristics.

In line with previous findings [5,7], our results showed that body weight was significantly under-reported and body height significantly over-reported. As a result, BMI was significantly under-reported. The under-reporting of body weight and over-reporting of height is understandable, considering the fact that being tall and slim is seen as ideal in Western society [26,30]. Also, if subjects weighed themselves at home with less clothing then the clothing they were wearing during the measurement, this could have contributed to the under-reporting of body weight. It is also possible that overweight subjects are less likely to weigh themselves and consequently do report their body weight with less accuracy [31].

The under-reporting of BMI in this study led to the under-reporting of obesity prevalence and the over-reporting of the overweight prevalence, the latter being due to the fact that our population consisted of only overweight and obese subjects. However, the overall prevalence of overweight (overweight and obesity combined) was under-reported in our population. As found in other studies [8,10] and in line with the aforementioned, the under-reporting of BMI was significantly greatest among the heavier subjects. In contrast with previous results [5,32,33], the reporting of body weight and height was not more biased in older subjects than in younger subjects.

We observed that WC was over-reported, especially in males, heavier subjects and less tall subjects. This was an unexpected finding, as under-reporting of WC has been consistently found in most other studies [20,21,34]. However, a slight over-reporting of WC has been observed in postmenopausal women aged 55 to 69 years [14]. Explanation for the over-reporting of WC is unclear. It has been suggested that subjects find it hard to measure their WC accurately [20], but there is no evidence to suggest that this would lead to an over-reporting of WC. Subjects may not have held the tape in horizontal position while measuring [14], may not have placed the measuring tape tight enough around their waist or may have measured their WC inadvertently at another, larger site than at the midpoint because of difficulty identifying this point. Also, subjects may have measured their WC at the end of an inhalation when their waist is being pulled out, instead of at the end of an exhalation.

Our finding that low SES subjects under-reported their body weight and BMI to a greater extent than those from a high SES is in concordance with previous findings [24,33]. In addition, it has been suggested that women who had a higher family income were more aware of their current weight and therefore more correctly self-reported their body weight [35], probably as they may have more access to weight loss programs and diet foods [6]. In line with the latter, low SES subjects will be less likely, due to their lower incomes, to buy an accurate weighing scale that is relatively expensive.

We also observed that subjects from a high SES over-reported their WC, whereas low SES subjects did not. If this finding may be interpreted as low SES subjects being more likely to under-report their WC than high SES subjects, it is in line with the under-reporting in these subjects of body weight and BMI to a greater extent than high SES subjects. It would have been interesting to also study the effect of income on the bias in the self-report of anthropometrics, as this is another socioeconomic variable with which SES can be conceptualized. However, we had many missing values for income, whereas education level was only missing for two subjects.

Except from the significant association between smoking status and the bias in self-reported body weight which is in line with a previous finding in men [26], none of the other health-related variables showed significant independent associations with the difference between measured and self-reported anthropometrics. Future studies including health-related variables are needed in order to get more insight into the association of these variables with bias in self-reported anthropometrics.

Although the self-reporting of body weight, height, BMI and WC was biased, the mean differences between measured and self-reported anthropometrics were low and the ICC's high, suggesting on average a high degree of accuracy of self-reported anthropometrics. Moreover, the percentages of agreement (and kappa's) pointed to a rather accurate classification of overweight, obesity and of increased WC. Therefore, the assessment of the prevalence of overweight/obesity in this overweight population could be done with reasonable accuracy. Recent evidence suggests that misclassification of self-reported BMI results in overestimated associations between overweight/obesity and concomitant morbidity [36,37]. Consequently, caution is necessary when assessing the prevalence of overweight-related health conditions based on self-reported anthropometrics.

The limits of agreement suggest that the individual self-reporting was less accurate. Consequently, self-reported anthropometrics are less suitable for the identification of overweight and especially obese individuals in our population. More insight into the characteristics of over-reporters and under-reporters will contribute to the accuracy of the assessment of the prevalence of overweight and obesity on an individual level.

Several limiting points need consideration. First, as our population consisted of overweight employees, the generalization of our results is limited. It would be interesting to know to what extent (non-employed) overweight/obese subjects misreport their anthropometrics compared to (non-employed) subjects with a normal weight (i.e., 18.5≤BMI < 25 kg/m^2^).

Second, our subjects had just started to participate voluntarily in a weight control program and therefore may have been well aware of their current weight (and height) [38], resulting in only a slight misreporting of body weight and height.

Third, we tried to minimize measurement errors in body weight, height and waist circumference by strict adherence to standard protocols and by using reliable measuring devices. However, during the six months in which body weight and height were assessed in all subjects, the two weighing scales and stadiometers that we used for these measurements were not calibrated, since it concerned standard commercially available devices. Calibration of both weighing scales, approximately one year after the measurement period, however, yielded no deviations.

Fourth, one may argue whether body weight and WC were self-assessed instead of self-reported, as at the appropriate questions in the questionnaire subjects were asked to weigh themselves and to measure their WC using the tape measure and instructions for use. However, we did not ask whether subjects indeed did weigh themselves or measured their WC prior to filling out the questionnaire. This may have produced some bias in the results, as self-assessed measures will be more accurate than self-reported ones.

Finally, our sample size is relatively large, yielding large statistical power. Therefore, we need to be cautious when interpreting our results; a statistical significant difference does not imply that this difference is of any clinical importance. Although measured weight and measured height showed significant independent associations with the accuracy of self-reported body weight and BMI, the regression coefficients were very small. Thus, the clinical significance of these findings may be limited.

## Conclusion

In conclusion, the results of this study suggest that although the self-reported anthropometrics are biased, self-reported BMI and WC are satisfactorily accurate for the assessment of the prevalence of overweight or obesity and of increased WC in an overweight working population. As self-reporting of anthropometrics can be done relatively easy and at low costs, it could be a useful tool to assess the overweight status in populations.

The individual self-reporting of body weight, height and BMI is less accurate, especially in heavier, less tall, low SES and non-smoking subjects. The self-reporting of WC is especially less accurate in males, younger subjects and subjects with a lower measured WC. Results for these subgroups should therefore be interpreted with caution. Also, due to the large power of our study, the clinical significance of our statistical significant findings may be limited.

Further research on the accuracy of self-reported anthropometrics and factors possibly affecting this accuracy in the general population are needed to get more insight into this issue.

## Competing interests

The authors declare that they have no competing interests.

## Authors' contributions

JCD participated in conception and design of the study, acquisition of data, analysis and interpretation of data and drafted the manuscript. MFW participated in the conception and design of the study, the acquisition of data and critically revised the manuscript. IJMH participated in the conception and design of the study and critically revised the manuscript. JWRT gave statistical advice and critically revised the manuscript. WM obtained funding, supervised the study, and critically revised the manuscript. All authors read and approved the final manuscript.

## Pre-publication history

The pre-publication history for this paper can be accessed here:



## Supplementary Material

Additional file 1 **Average intra-class correlation coefficients (95% CI) by sex, and by age, BMI groups, SES groups, smoking status, medication use and frequency of weighing oneself groups.** Click here for file
