# Supplementary material for: Accuracy of self-reported body weight, height and waist circumference in a Dutch overweight working population
Source: BMC Med Res Methodol. 2008 Oct 28;8:69. doi: 10.1186/1471-2288-8-69 (PMC2605752; doi:10.1186/1471-2288-8-69)
Supplement: Additional file 1 — Average intra-class correlation coefficients (95% CI) by sex, and by age, BMI groups, SES groups, smoking status, medication use and frequency of weighing oneself groups. [file 1471-2288-8-69-S1.doc]

**Additional file 1**

*Average intra-class correlation coefficients (95% CI) by sex, age, BMI status, SES,*

*smoking status, medication use and frequency of weighing oneself.*

| Anthropometrics | ICC | 95% CI | ICC | 95% CI |
| --- | --- | --- | --- | --- |
|  | **Males (N=864)** | | **Females (N=434)** | |
| Body weight (kg) | 0.99 | 0.99 to 0.99 | 0.99 | 0.99 to 1.0 |
| Body height (cm) | 0.99 | 0.99 to 0.99 | 0.99 | 0.98 to 0.99 |
| BMI (kg/m^2^) | 0.98 | 0.98 to 0.98 | 0.99 | 0.99 to 0.99 |
| WC (cm)* | 0.96 | 0.94 to 0.97 | 0.91 | 0.86 to 0.94 |
|  | **Low age (N=649)** | | **High age (N=649)** | |
| Body weight (kg) | 0.99 | 0.99 to 1.0 | 1.0 | 0.99 to 1.0 |
| Body height (cm) | 0.99 | 0.99 to 1.0 | 0.99 | 0.99 to 0.99 |
| BMI (kg/m^2^) | 0.99 | 0.98 to 0.99 | 0.98 | 0.98 to 0.99 |
| WC (cm)* | 0.96 | 0.95 to 0.97 | 0.95 | 0.93 to 0.97 |
|  | **Low BMI (N=861)** | | **High BMI (N=437)** | |
| Body weight (kg) | 0.99 | 0.99 to 0.99 | 0.99 | 0.99 to 0.99 |
| Body height (cm) | 0.99 | 0.99 to 0.99 | 0.99 | 0.99 to 0.99 |
| BMI (kg/m^2^) | 0.92 | 0.92 to 0.94 | 0.98 | 0.97 to 0.98 |
| WC (cm)* | 0.92 | 0.89 to 0.94 | 0.95 | 0.93 to 0.97 |
|  | **Low SES (N=513)** | | **High SES (N=784)** | |
| Body weight (kg) | 1.0 | 0.99 to 0.99 | 1.00 | 0.99 to 1.00 |
| Body height (cm) | 0.99 | 0.99 to 0.99 | 0.99 | 0.99 to 0.99 |
| BMI (kg/m^2^) | 0.99 | 0.98 to 0.98 | 0.99 | 0.98 to 0.99 |
| WC (cm)* | 0.95 | 0.92 to 0.96 | 0.96 | 0.95 to 0.97 |
|  | **Smoking (N=193)** | | **Non-smoking (N=1103)** | |
| Body weight (kg) | 0.99 | 0.99 to 1.00 | 1.00 | 1.00 to 1.00 |
| Body height (cm) | 0.99 | 0.99 to 1.00 | 0.99 | 0.99 to 0.99 |
| BMI (kg/m^2^) | 0.98 | 0.99 to 0.99 | 0.99 | 0.98 to 0.99 |
| WC (cm)* | 0.93 | 0.87 to 0.97 | 0.96 | 0.95 to 0.97 |
|  | **Medication use (N=218)** | | **No medication use (N=1031)** | |
| Body weight (kg) | 1.0 | 0.99 to 1.0 | 1.0 | 0.99 to 1.00 |
| Body height (cm) | 0.99 | 0.99 to 1.00 | 0.99 | 0.99 to 0.99 |
| BMI (kg/m^2^) | 0.99 | 0.98 to 0.99 | 0.98 | 0.98 to 0.99 |
| WC (cm)* | 0.98 | 0.95 to 0.99 | 0.95 | 0.94 to 0.96 |
|  | **Low frequency of weighing oneself (N=671)** | | **High frequency of weighing oneself (N=627)** | |
| Body weight (kg) | 0.99 | 0.99 to 1.00 | 1.00 | 1.00 to 1.00 |
| Body height (cm) | 0.99 | 0.99 to 0.99 | 0.99 | 0.99 to 0.99 |
| BMI (kg/m^2^) | 0.98 | 0.98 to 0.98 | 0.99 | 0.99 to 0.99 |
| WC (cm)* | 0.96 | 0.95 to 0.97 | 0.95 | 0.92 to 0.96 |

* Results on WC are based on a sub-sample of 250 subjects (176 males and 74 females; low age group [N=125], high age group [N=125]; low BMI group [N=163], high BMI group [N=87]; low SES group [N=99], high SES group [N=151]; smoking [N=36], non-smoking [N=212]; medication use [N=33], no medication use [N=209]; low frequency of weighing [125], high frequency of weighing [123]).
